# The Influence of History of Severe Periodontitis on Estimated Long-Term Marginal Bone Loss around Implants Restored with Fixed Segmented Full-Arch Rehabilitation

**DOI:** 10.3390/jcm12206665

**Published:** 2023-10-21

**Authors:** Pablo Galindo-Moreno, Andres Catena, Lucia Lopez-Chaichio, Tiago Borges, Francisco O’Valle, Laura Torrecillas-Martínez, Miguel Padial-Molina

**Affiliations:** 1 Department of Oral Surgery and Implant Dentistry, School of Dentistry, University of Granada, 18071 Granada, Spain; mipadial@ugr.es; 2 Instituto de Investigación Biosanitaria ibs.GRANADA, 18071 Granada, Spain; fovalle@ugr.es (F.O.);; 3 Department of Experimental Psychology, School of Psychology, University of Granada, 18071 Granada, Spain; 4 Private Practice, 23650 Jaen, Spain; 5 Centre of Interdisciplinary Research in Health, and Faculty of Dentistry, Universidade Católica Portuguesa, 3504-505 Viseu, Portugal; tborges@ucp.pt; 6 Department of Pathology and IBIMER, School of Medicine, University of Granada, 18071 Granada, Spain; 7 Private Practice, 18008 Granada, Spain

**Keywords:** peri-implantitis, marginal bone loss, periodontitis, dental implants

## Abstract

The aim of this study was to analyze the long-term marginal bone level (MBL) of implants supporting fixed full-arch restoration in patients who had previously lost their dentition due to severe periodontitis. This retrospective study included 35 patients in whom 342 implants with internal tapered conical connections were placed. MBL was analyzed radiographically over time and a long-term estimation of MBL was calculated. A mixed linear model with abutment height, graft, diameter and location (maxilla/mandible) as factors and gender, age, implant length and prosthetic variables as covariates was used to evaluate the influence on MBL. MBL in these patients showed an estimator of predictions at 4108 days after loading of −0.307 mm, SE = 0.042. Only 0.15% of implants were radiographically affected with MBL of 3 mm or more. The mixed linear model results showed a main effect of the type of opposing dentition, gender, implant diameter, and abutment height. Particularly, an abutment height of 1 mm had associated larger MBL than the remaining heights. Thus, it can be concluded that dental implants restored with fixed segmented full-arch rehabilitation in patients with a history of severe periodontal disease do not suffer important marginal bone loss if some specific factors are considered, mainly the use of long transmucosal abutments (≥2 mm).

## 1. Introduction

Implant therapy has become the gold standard to substitute lost teeth. Despite many different causes of tooth loss, caries and periodontal disease are the most frequent entities that determine tooth extraction. In fact, in developed countries, periodontal diseases affect more than 65% of the adult population over 65 years old [1], leading to complete edentulism in severe cases. Considering that periodontal diseases affect gingiva and supportive structures of the teeth, supportive elements of implants could be affected by similar diseases as well. So, by analogy, periodontally compromised patients would be more prone to marginal bone loss (MBL) and implant loss than those who lost their teeth due to any other etiology. Although some reviews and meta-analysis found lower implant survival only for aggressive forms of periodontal disease [2,3,4,5], a marked trend is described in the literature pointed towards higher pocket probing depths (PPDs) [6] and marginal bone loss [7,8,9] in implants placed in periodontally compromised patients when compared to implants placed in periodontally healthy patients. Nevertheless, the opposite has also been reported [10].

Curiously, previously published meta-analyses show remarkable data to consider. In patients with aggressive forms of periodontal disease, MBL around implants is higher than in healthy patients or in those with chronic periodontitis; but there are no differences between groups in terms of secondary parameters, such as pocket probing depth, plaque index, gingival index or clinical attachment level [2,3,4]. However, the data from these meta-analyses point out the importance of the follow-up period. One of the studies showed less MBL in patients with a previous history of chronic periodontitis than in healthy patients in short and medium follow-ups [2]. Meanwhile, Kim and Sung reported no differences in MBL around implants in healthy patients versus those in patients with chronic periodontitis, but found differences between patients with aggressive periodontitis (higher MBL) and patients with chronic periodontitis or who were periodontally healthy only in long-term follow-ups [4]. On the contrary, Monje and coworkers reported a similar behavior in group comparisons but found no influence of the follow-up period on the final outcomes [3]. Noteworthily, these three reviews analyzed almost the same studies but the results are not completely concordant, and the evidence is inconclusive. These differences could be explained by several reasons. Firstly, the evaluation of the studies to be included in the meta-analyses can lead to potentially subjective interpretation of data; and secondly, the heterogeneity of the implants evaluated in the studies can lead to different final outcomes.

A previous history of periodontitis could not play such an important role in the appearance of peri-implantitis, but in its progression. In this sense, a recent study did not find a statistically significant association between periodontitis stage or grade and the prevalence of peri-implantitis. However, in that study, when peri-implantitis was diagnosed, either a relationship between the grade of periodontitis and the severity of peri-implantitis could be found or an increased occurrence of implant failure was evidenced [11].

Moreover, prosthetic rehabilitation plays a major role in the final outcome of the treatment. Nowadays, prosthetic options and possibilities available in the armamentarium to rehabilitate fully edentulous jaws are nearly limitless. While removable prosthesis still remains as an alternative, mainly because of cost-related issues [12,13], most patients do not get satisfaction as high as those with implant-supported fixed dentures [14]. In the scope of fixed prosthesis, the amount, distribution and angulation of implants placed to restore full-arch cases may vary. In a wide proportion, these are patients who had lost their teeth together with their supporting tissues, which means that bone availability can be an issue. In these cases, bone grafting is the only alternative in order to (a) place implants in a correct three-dimensional position, where the load is directed along their long axis so the occlusal stress is evenly distributed over the surrounding bone [15] and the probability of prosthetic complications such as screw loosening/screw fracture is minimized [16]; (b) fabricate screw-retained restorations, eliminating the risk of leaving excess cement in the sulcus, which could potentially lead to an inflammatory reaction around the fixture [17] and (c) be able to divide the full arch restoration into sections/bridges, facilitating future retrievability and repairs. This treatment choice is not exempt from difficulties. Despite the bone-grafting procedure if needed, at least 7–8 implants are needed [18], with the consequent treatment time, effort in planification and surgical intervention, cost and patient discomfort. In addition, once these implants are osseointegrated, many prosthesis-related factors such as the new occlusal scheme [19,20], the resulting crown-to-implant ratio [21], or the abutment selection [22], could influence crestal bone changes and, hence, the long-term success of the rehabilitation.

In this sense, most of the studies analyzing the behavior of implants in periodontally compromised patients are conducted on single crowns, fixed partial restorations, or overdentures. However, outcomes worsen dramatically in patients with fixed full-arch restorations. Although Kwon and colleagues have suggested a 78.3–98.9% of implant survival for full-arch fixed hybrid prostheses [23], information regarding implants supporting a fixed full-arch rehabilitation in patients with a previous history of periodontitis is already quite limited [24]. Thus, the aim of the present study was to analyze the long-term marginal bone levels around implants supporting screw-retained fixed full-arch restorations in patients who had lost their dentition due to severe periodontal disease. Our hypothesis was that some parameters, including the height of the transmucosal abutment, would influence the marginal bone loss around implants in these patients.

## 2. Materials and Methods

### 2.1. Study Population

The reporting of this study follows the STROBE-EQUATOR guidelines.

This retrospective case-series study is part of the protocol approved by the Ethics Committee for Human Research of the University of Granada (487/CEIH/2018). The protocol was approved to conduct a retrospective radiographic study on MBL in a series of patients treated with a specific type of dental implant (AstraTech TX, now part of Dentsply Implants, Mölndal) under the same clinical protocol. Data were collected from patients referred to a faculty practice of the University of Granada. Short-term MBL analysis of the whole pool of patients has been recently published, including both partially or totally edentulous patients [25]. Out of the whole set of patients, for this study, only those patients treated between 2007 and 2018 who underwent the extraction of all their teeth in the maxilla, mandible or both jaws at least 8 weeks before as a consequence of severe periodontitis, currently known as Stage 4, and received a fixed implant-supported full-arch segmented rehabilitation were included. For the current analysis, the follow-up period since implant placement and loading was not limited so that all data were analyzed. The MBL analysis after 5 years of loading in 19 fully edentulous patients in the maxilla, the mandible or both restored with 160 implants supporting fixed implant-supported full-arch segmented rehabilitation has been previously reported [26].

Subjects with any medical condition such as non-treated periodontal disease in the opposing arch (if present), any kind of sinus pathology in patients that required grafting of these areas, vertical or horizontal bone augmentation, any disease or intake of medication known to alter bone metabolism or previous radiotherapy in the oral environment were excluded from the study.

### 2.2. Surgical and Restorative Procedures

As reported elsewhere [25], all surgical procedures were conducted in a dental chair under local anesthesia (Ultracain^®^, Aventis Inc., Frankfurt, Germany). All the surgeries were conducted by the same experienced oral surgeon (P.G.-M.). A full-arch mucoperiosteal incision was performed in the middle of the alveolar crest, including the keratinized mucosa when it was present, with two small vertical releases in the posterior areas. A full-thickness flap was elevated in the vestibular and lingual/palatal areas, to expose the whole alveolar crest. In those patients with limited vertical bone height in the posterior upper maxilla (less than 8 mm), sinus augmentation procedures were performed. No other kinds of grafting techniques, such as vertical or horizontal augmentations, were conducted in these patients. A total of at least 8 implants were placed per arch. All implants were placed with the implant shoulder at the level of the bone crest. Only one clinical case was restored with 6 inferior implants because two implants failed before loading and the patient refused to have them replaced. In order to design a convenient occlusal scheme, according to Misch and Silc’s recommendations [18], when possible, implants were placed in the location of central incisors, canines, first premolars and first molars. This scheme allows for a fixed full-arch rehabilitation with at least 12 crowns, segmented in four 3-unit bridges if prosthetically and esthetically convenient. All implants analyzed in this study were OsseoSpeedTM AstraTech TX implants with internal tapered conical connection (now part of Denstply Implants, Mölndal, Sweden), of 3.5, 4.0, 4.5 and 5 mm in diameter and 6, 9, 11, 13 and 15 mm in length.

After the implant’s installation, all patients were prescribed a pharmacological regimen of amoxicillin/clavulanic acid (875/125 mg, TID for 7 days) or, if allergic to penicillin, clindamycin (300 mg, TID for 7 days), and anti-inflammatory medication (Ibuprofen 600 mg every 4–6 h as needed, with a maximum of 3600 mg/day). Analgesic medication was prescribed if needed (metamizole 550 mg). Sutures were removed one week post-op and healing was evaluated biweekly until second surgical stage.

The second surgical stages were conducted after 8 weeks, or after 6 months if maxillary sinus augmentation had been performed, and, 2 weeks later, trans-epithelial abutments were placed. Straight Lilac or Aqua uni-abutments (Dentsply Implants, Mölndal, Sweden) were used in all cases to connect the implants to the screwed restoration. The uni-abutments were 1, 2, 4 or 6 mm in height according to the thickness of the peri-implant mucosa. In the same clinical appointment when the trans-epithelial abutments were placed, final impressions were taken using prosthetic uni-abutment transfers. After impressions and try-ins, screw-retained metal–ceramic fixed implant-supported prostheses were delivered between 4 and 6 weeks after the second stage surgery. Then, patients were recommended to follow a regular implant maintenance program with daily care by tooth brushing, use of irrigators, and professional follow-up annually.

### 2.3. Radiographic Evaluation of MBL

Digital panoramic radiographs (Instrumentarium 700 3D module, Finland) were obtained at the time of treatment planning, after implant placement (baseline), at restoration delivery, at 1 month, 6 months, 12 months after delivery and every time the patient came for follow-up after that (normally every year). Images were then exported as DICOM files and evaluated in the ImageJ software (NIH, Bethesda, MD, USA). Before any study images were evaluated, as explained in a previous study [26], an intra-examiner calibration exercise was conducted for a single independent calibrated examiner (M.P.-M.). The calculated intraclass correlation coefficient was 0.892. For the determination of marginal bone level, the calibrated examiner made linear measurements on each panoramic radiograph from the most mesial and distal points of the implant platform to the crest of the bone. The magnification of the radiographs was corrected in accordance with the clinical data available for each implant, particularly the width at the implant shoulder.

### 2.4. Additional Data Recorded

Clinical data recorded included age at the time of implant placement, gender, implant location (maxilla or mandible), position, length, and diameter (≤3.5 mm or ≥4.00 mm), need for a sinus graft, and height of the abutment placed. Additional data included type of opposing dentition to each one of the bridges (natural dentition, removable denture, implant-supported fixed bridge, teeth-supported fixed bridge) as well as to the whole arch restoration (natural dentition, mixed, implant-supported fixed bridge, implant-supported overdenture), height of the crown, number of implants per bridge, and number of crowns per bridge. All patients were smokers or former smokers, so this factor was not considered in the current analysis.

### 2.5. Statistical Analysis

A total of 342 implants, placed in 35 patients, were evaluated in this retrospective study. In order to estimate the MBL in the mesial and distal aspects of the implants as a function of time, we estimated the MBL at 4108 days from loading. We selected 11.25 years as the follow-up (4108 days) for the estimated outcomes since this was the longest interval of time after loading measured for some of the included patients (a total of 8 implants). This allowed us to homogenize our statistical analysis in terms of time of follow-up. Our analysis protocol was the following: firstly, we obtained the parameters for the power law expressing the actual relationship between MBL and follow-up time for each implant; secondly, we estimated the predicted MBL at 4108 days after loading; and, thirdly, these estimations were submitted to the mixed linear model.

No differences were observed between mesial and distal bone levels; thus, the average of mesial and distal measures was used to conduct the statistical analyses. We used a mixed linear model with gender, abutment height, graft, diameter, location, type of opposing dentition and opposing arch as factors, and age, crown height, crown-to-implant ratio, implants per bridge, crowns per bridge, bridge ratio and implant length as covariates. The covariance matrix was selected using the Schwarz Bayesian Information criterion (BIC). We assessed scaled identity, diagonal, Huynh–Feldt, and first-order analytic factor structures for this matrix. The final covariance structure selected was Diagonal (BIC = 687.635). In order to check the sensitivity of our estimations and our statistical approach, we performed the same mixed model using the last measurement of MBL. Again, the Diagonal covariance structure was used.

## 3. Results

Frequencies and descriptive statistics of the study variables are displayed in Table 1 and Table 2.

The main variable of this study, MBL in implants placed in patients with a previous history of periodontitis (Table 3), showed an estimator of predictions at 4108 days after loading of −0.307 mm, SE = 0.042. No implant was lost during the follow-up of this study. Only 0.15% of implants were radiographically affected with MBL of 3 mm or more, so, if radiographical success is established at 2 mm of MBL, as it has been classically defined [27], our series has a 95.3% probability of success. Moreover, 18.9% of implants showed a cut-point of MBL higher than 0.5 mm.

**Figure 1:**
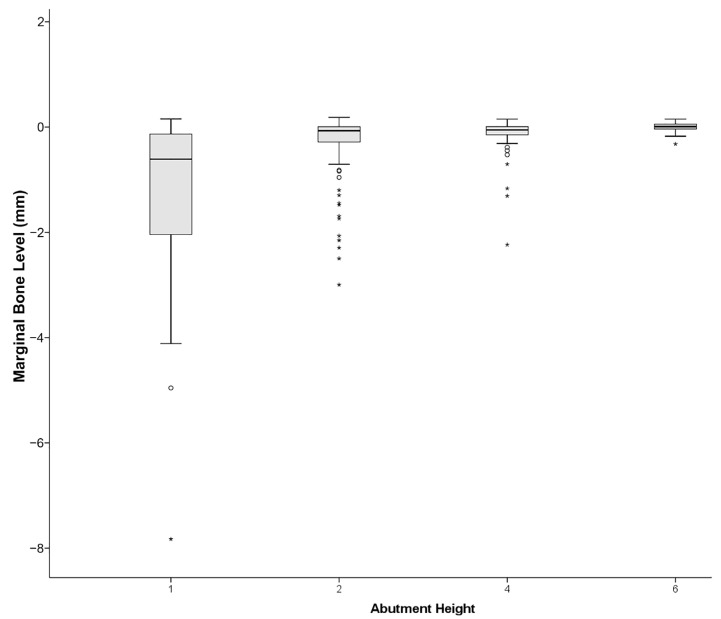
Box-and-whiskers representation of marginal bone level as a function of the abutment height. °: mild outliers; *: extreme outliers.

**Figure 2:**
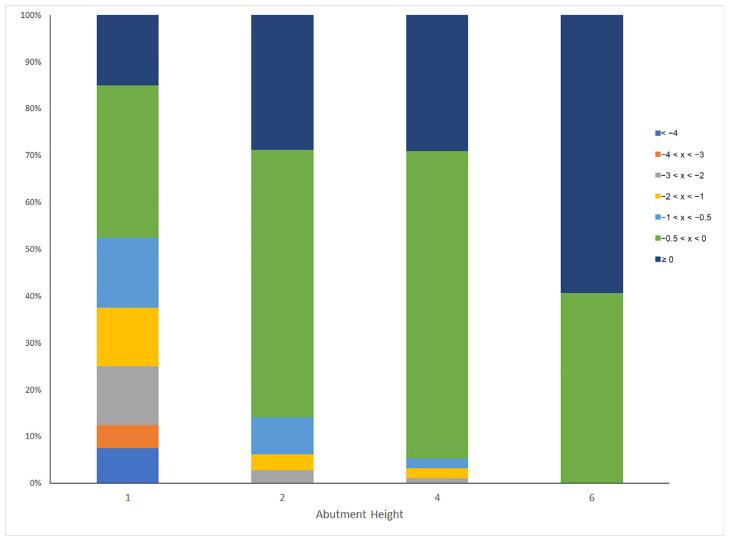
Percentage of implants with different levels of marginal bone loss in each category of abutment height.

The mixed linear model results indicated that gender, age, abutment height, type of opposing dentition and diameter were related to MBL estimates. No other variables showed a significant influence in the MBL in our sample. The analysis showed a main effect of abutment height, F(3,27) = 10.76, *p* < 0.001. Detailed analysis of this effect showed that when the abutment height was 1 mm, it was associated with a larger MBL (−0.593, SE = 0.12), compared to the remaining heights (−0.274, SE = 0.11, −0.192, SE = 0.11, and −0.139, SE = 0.13, for abutment heights of 2, 4 or 6 mm, respectively) (Figure 1). The influence of the abutment height on MBL by the percentage of implants affected in each category is expressed in Table 4 and Figure 2. We also observed an effect of diameter, F(1,25) = 27.15, *p* < 0.001, the MBL being larger in the narrower implants (−0.401, SE = 0.11 and −0.197, SE = 0.11, respectively). The type of opposing dentition also yielded an effect, F(3,185) = 5.389, *p* = 0.001, the opposing mixed prothesis and implant-supported overdentures being the ones that were associated with larger MBL (−0.012, SE = 0.17, −0.079, SE = 0.15, −0.402, SE = 0.12, and −0.705, SE = 0.36, for natural dentition, implants-supported fixed bridge and mixed and implant-supported overdentures, respectively). Women had lower levels of MBL than men, F(1,42) = 14.76, *p* < 0.001 (−0.214, SE = 0.11, and −0.384, SE = 0.11, for women and men, respectively). Finally, we detected that as the age increases, the MBL tends to be lower, at a rate of 0.0073 by year. None of the remaining variables yielded significant effects. The effects were the same for the mesial and the distal aspects.

For our sensitivity analysis, we used the MBL measured at the end of the follow-up period, which was different for each patient. We observed again a main effect of abutment height, F(3,22) = 11.59, *p* < 0.001, diameter, F(1,31) = 25.21, *p* < 0.001, type of opposing dentition, F(3,187) = 6.36, *p* < 0.001, and gender, F(1,48) = 9.82, *p* = 0.003. Detailed analysis of these effects yielded the same differences we observed on the MBL estimates at 4108 days.

**Figure 3:**
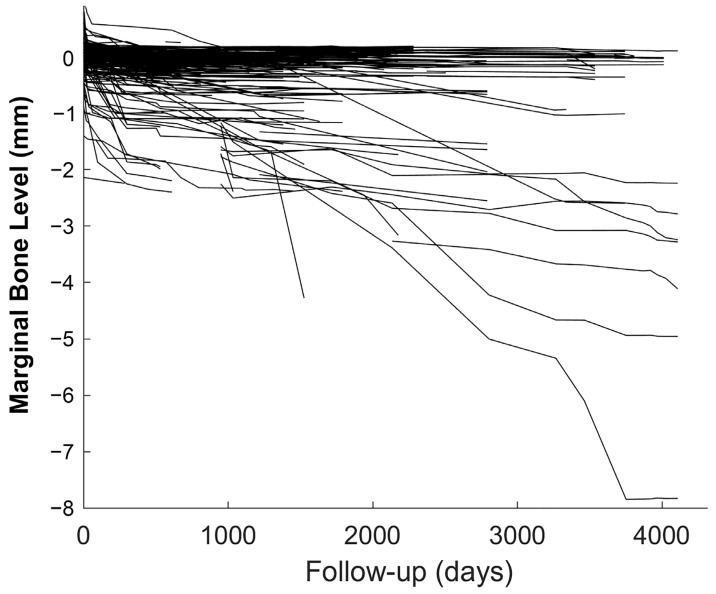
Marginal bone level as a function of time since loading.

**Figure 4:**
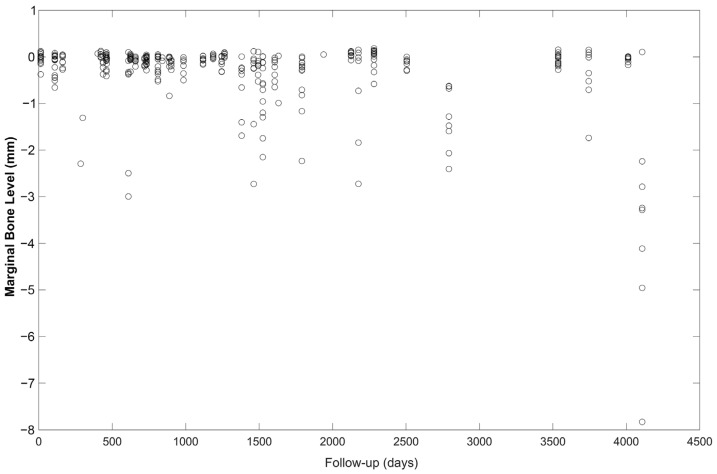
Representation of the estimated marginal bone level as a function of the time elapsed from loading.

Due to the singularity of the sample, where all our patients have had severe periodontitis and the loss of many or even all of their teeth because of it, a major aim in our study was to determine and estimate the progression of MBL as a function of time since loading. In this sense, the course of the MBL as a function of time from loading is showed in Figure 3, and the representation of the estimated MBL as a function of the time elapsed from loading is displayed in Figure 4.

**Figure 5:**
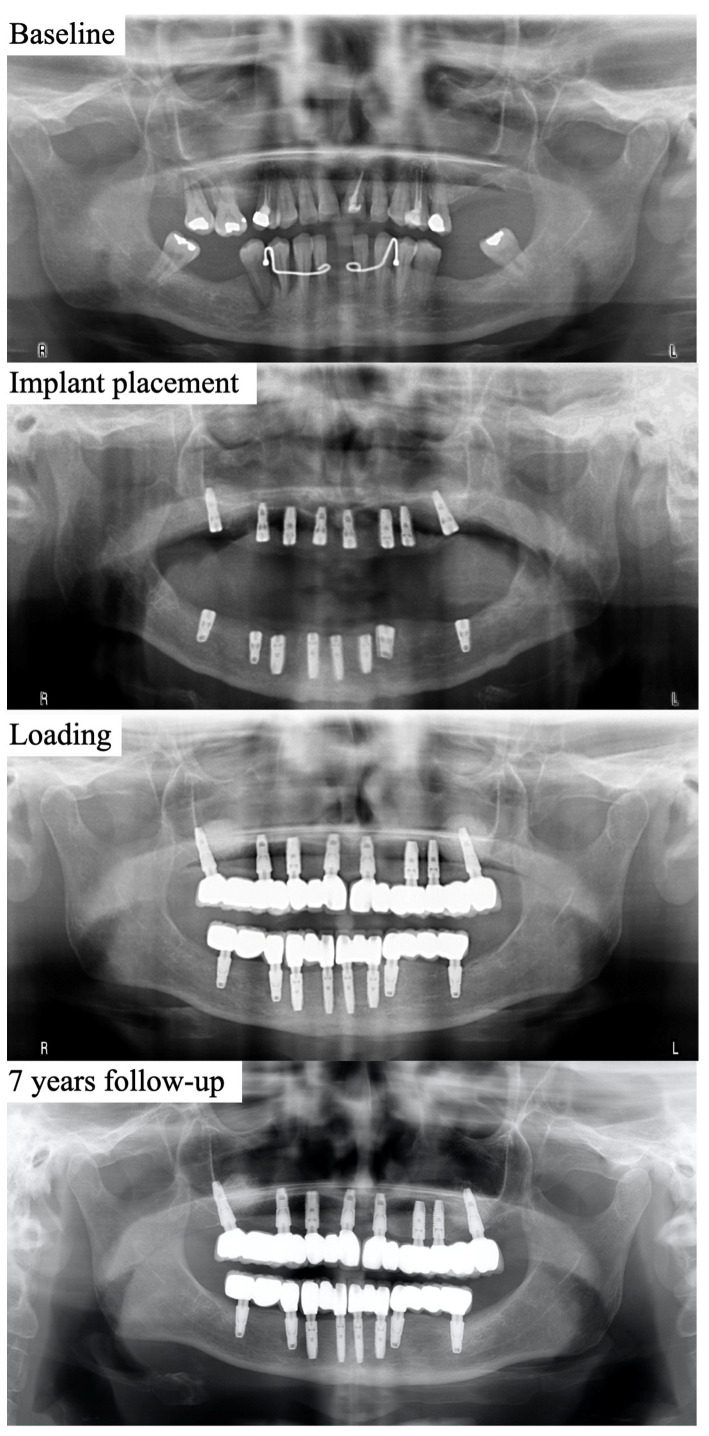
Representative series of radiographs from an included case.

Figure 5 represents an illustrative case of the evolution over time of a successful case.

## 4. Discussion

The aim of the present study was to analyze the long-term marginal bone level in implants placed in patients who had lost all their teeth due to severe periodontitis. Implants were restored with fixed full-arch implant-supported rehabilitations. Our results show, in a large sample of implants (342) and patients (35), that the estimated mean MBL after 11 years of follow-up will be −0.307 mm (SE = 0.042). In the mesial aspect the MBL was −0.297 (SE = 0.04) and in the distal aspect it was −0.317 (SE = 0.04). Only 0.15% of implants presented radiographically 3 mm or more of bone loss from the most coronal aspect of the implant. If radiographical success is established at 2 mm of MBL, as it has been classically defined, our series had 95.3% success. Moreover, if the cut point is set at 0.5 mm or more, the percentage of implants affected is only 18.9%, much less than the 24.9% found by Derks and coworkers [28]. Our results are not in accordance with those by Mengel and coworkers either. They recorded maxillary marginal bone loss of 0.75 ± 0.66 mm after 5 years, and 1.50 ± 1.45 mm after 20 years. Despite their results, they suggest that implants placed in severely periodontally compromised patients could be successfully managed in the long term [29], which we agree with.

Data presented in this manuscript suggest that a previous history of severe periodontitis does not condition the evolution of dental implants in terms of development of marginal bone loss, which finally could end up in peri-implantitis. It can be understood, once again, that if the implants are placed in a correct three-dimensional position, one key factor in the development of marginal bone loss is the height of the prosthetic abutment [22], due to its role in 1. the adaptation of the supracrestal tissues surrounding implants once these are exposed to the oral environment [30], and 2. the distancing of the inflammation-inducing gap between the prosthetic crowns and the abutment from the bone [31]. It has been suggested that since the height of the abutment is related to the thickness of tissue, this later parameter is the real key factor in the preservation of peri-implant bone, a 2 mm thickness of peri-implant tissue being enough to prevent bone loss [32,33]. However, recent manuscripts have evidenced bone preservation around implants with thin mucosa restored using long abutments [34] and, on the contrary, peri-implant bone resorption when thick mucosa was present but 1 mm abutments were inserted [35,36]. The latter studies clearly highlight the important role of the abutment height in peri-implant bone preservation in comparison with the thickness of the mucosa.

It has been also suggested that keratinized supra-crestal tissue has a major role in peri-implant bone preservation, studies describing how less than 2 mm of keratinized tissue width would promote soft tissue inflammation [37]. Thus, a reduced peri-implant keratinized mucosa can be associated with increased marginal bone loss and the prevalence of peri-implantitis [38]. On the contrary, Ravidà and coworkers in a recent meta-analysis remarked that implants associated with <2 mm keratinized mucosa width did not exhibit increased marginal bone loss, soft tissue recession nor probing depth compared to implants with ≥2 mm of keratinized peri-implant mucosa. They conclude that the quality of evidence supporting keratinized mucosa as a risk factor for peri-implant bone loss and establishment of disease and the 2-mm cut-off point used in the literature is low at best [39]. In the present study, the role of the peri-implant soft tissues could not be examined. However, our radiographic results showed that a 2 mm abutment height was enough to prevent marginal bone loss regardless of other parameters, as previously reported in different series of patients [22,25,40,41,42]. It can be assumed that in prostheses supported by multiple implants, the farther the prosthetic crowns and the prosthetics gap are from the bone, the greater the bone preservation.

In accordance with this, Wang and coworkers established that tissue level implants placed in patients with uncontrolled periodontitis maintained the crestal bone similar to implants placed in non-periodontitis patients, while their natural teeth showed a higher bone loss in comparison to the healthy patients [43]. Plausible explanations for this clinical finding could be: 1. Tissue level implants have a polished collar that reduces bacterial adhesion compared to rough surfaces. 2. The increased distance between the bone crest and the crown margin or the implant-abutment interface. In this sense, it has even been suggested that the vertical extension shows a higher relevance than the horizontal mismatching in terms of bone preservation around the neck of the implant [44]. Thus, not only is the mismatching important, but also the height of the abutment.

In terms of survival, our results in terms of actual data (patients with long follow-up) and in terms of the estimate to 11 years of evolution are really far from those previously reported in the literature. Kwon and coworkers have suggested a 78.3–98.9% probability of implant survival for full-arch fixed dental hybrid prostheses [23] while Papapyridakos and coworkers reported a peri-implant bone loss greater than 2 mm in 20.1% of the implants after 5 years and 40.3% after 10 years [45] in the same type of prosthesis. One of the main reasons that could explain these differences is the type of fixed restorations: hybrid prostheses versus implant-supported fixed bridges, where the ease for hygienic maintenance is completely different.

The present research also paid attention to the influence of the prosthetic design in the biological process of bone resorption around dental implants. The prosthetic design has multiple variables to take into consideration: (a) implant platform characteristics; such as the type of connection (external hex, internal hex, internal conical…) or the diameter of the implant, (b) implant–prothesis interphase or the connection of the prosthetic crown to the implant platform; it can be connected directly to the platform or using an intermediate abutment and it can be screw- or cement-retained, and (c) implant–prosthesis relationships, such as the crown-to-implant ratio, the crown-per-bridge ratio, and the length of the prosthetic span, among others. Aiming to achieve long-term stability and success, our reconstructions were built following concepts previously described in the literature [18]; internal conical-connection standard implants to minimize MBL where the platform already incorporates the “platform switching” concept, connected to a PFM framework through intermediate abutments to allow space for the establishment of the peri-implant soft tissues, avoid repeated screwing and unscrewing of the components to the implant, and ensure implant–abutment engagement. Finally, we used screw-retained restorations to avoid excess cement in the peri-implant surrounding tissues [17]. Additionally, by segmenting the restoration, the accuracy of the adjustment of the supra-structures to the abutment increases and future retrievability for maintenance or repairs becomes easier. Cantilevers were not used in our prosthetic design because of their negative effects on MBL [46].

This study has found that narrower implants present higher MBL. There are two important aspects that can be discussed to justify this finding. First, it could be hypothesized that when narrower implants are used, there is more bone supporting the implant neck and surrounding it. However, this parameter has not been specifically analyzed in this study; thus, narrower implants may have been used in this study in more compromised alveolar crests more prone to lose bone. This is particularly true if the thickness of the surrounding bone walls is reduced. Secondly, and more importantly, the implants being used in this study share abutment sizes for different implant diameters. Thus, in narrower implants, the horizontal dimensions of the platform switching are lower than in wider implants. As demonstrated by Canullo et al., this amount of mismatching has a direct effect on MBL [47]. Interestingly though, when the same mismatching is present, the MBL is comparable regardless of the implant and abutment diameter [48].

Finally, we cannot forget that these full-arch rehabilitations have been performed in patients who had a history of previous periodontitis, and that, as we have been highlighting, there is controversy about the final outcomes of implants in this kind of patient. Therefore, it is important to underline the relationship of these two sets of variables, periodontal and prosthetic.

Traditionally, clinical studies have reported that implants placed in periodontally affected patients show higher marginal bone loss in comparison with those placed in healthy patients. This led to consideration of a history of periodontal disease as a predisposing factor in the development of peri-implantitis [49]. However, some reviews offer several points for discussion on that statement, including the lack of properly randomized studies [50] or the high heterogeneity of the included manuscripts [5]. Thus, results from these and other meta-analyses and reviews must be considered with care because of several reasons:

1. Firstly, there is a probabilistic issue. It is easier to lose more bone and implants when there are more implants placed. In fact, there are some studies affirming that to have more than four implants is a risk factor to develop peri-implantitis [28]. Then, since patients with periodontal disease usually lose more teeth, they would carry more implants and would be at higher risk of complications.
2. It is very well known that periodontitis and peri-implantitis are pathological processes that begin with soft tissue inflammation. New theories support the idea that a pro-inflammatory profile could be a requisite needed to go from a symbiotic to a dysbiotic status, which would subsequently allow microorganisms to play a pathological role [51,52]. In addition, some microorganisms are even able to manipulate the host to “play their game” [53]. Regardless, the altered pro-inflammatory response profile to specific bacteria in periodontally compromised patients will continue even if all teeth are removed and replaced with implants. This is because some periodontophatogens are kept in the oral mucosa and tongue over time [54,55,56]. However, the extraction of infected periodontal teeth reduces the pathological bacterial load, and, consequently, the bacteria-promoted inflammation.
3. Periodontal disease and peri-implantitis follow a different pattern regarding bone resorption. It is well known that periodontal bone resorption usually affects most patients’ teeth leading to a pattern of horizontal bone resorption. In contrast, peri-implantitis usually affects only one implant and leads to a vertical bone resorption that evolves to a circumferential defect. This even happens with a different bone loss pattern in each implant when several implants are affected by peri-implantitis in the same patient. This is the reason why epidemiologically in periodontitis “patient level” is used but in peri-implantitis there is a need to introduce the “implant level” factor.
4. This “implant level” factor becomes more obvious when the high heterogeneity of implant designs is considered, which leads to a high heterogeneity in the studies presented in the literature. It is very well known that some features of the implants condition the final outcome in terms of marginal bone loss, as extensively discussed in the literature. For instance, there is the type of implant connection. In our previous studies, a previous history of periodontitis was a significant factor in the progression of marginal bone loss [57]. However, external and internal connection implants were analyzed. In the current study we have analyzed only internal conical connection implants and obtained really good outcomes in terms of MBL. So, the type of implant–abutment connection plays a major role in marginal bone loss, that can even be superior to that of a previous history of periodontitis.

This study has some limitations. Firstly, it is a case-series retrospective study. Although the sample size in terms of number of patients and implants could be considered adequate, the homogeneity of the sample cannot be controlled. Secondly, in this study, digitalized panoramic radiographies were used. Although the authors know that periapical radiographs have been described as the ideal technique to measure peri-implant MBL [58], numerous published studies also use panoramic radiographs with internal calibration, as we did in the current study. On the other hand, it is obvious that these radiographic retro-alveolar techniques (periapical radiographs) cannot be conducted in the atrophic maxilla, where the alveolar processes had been severely resorbed. Thirdly, this study lacks data regarding the clinical status of peri-implant soft tissues. Although an intense discussion can be established on the role of these data in retrospective radiographic studies to diagnose marginal bone loss [25], and consequently peri-implantitis, the truth is that the only diagnostic difference between mucositis and peri-implantitis is progressive marginal bone loss. This can only be demonstrated by imaging techniques. Finally, in this type of study, it is difficult to establish a control group for several reasons. First, it is not common to find patients who had lost all their teeth due to a non-periodontal-related condition. Second, in severely affected periodontal patients, extraction of all teeth is usually the most commonly selected treatment option, instead of trying to maintain teeth that may complicate the long-term prosthetic solution. In addition, although bone resorption was truly advanced in these patients, we decided to exclude patients in need of vertical or horizontal augmentation because grafting procedures at the level of the implant shoulder may influence the outcomes much more than if the graft is placed in the maxillary sinus. This does not necessarily imply that bone resorption because of the previous periodontitis process was not advanced. In fact, many of our patients were subjected to sinus graft.

## 5. Conclusions

Implants restored with fixed full-arch segmented rehabilitation in patients with a history of severe periodontal disease do not suffer important marginal bone loss, if some specific factors are taken care of. These key precautions, according to the current study, mainly include the use of long transmucosal abutments (≥2 mm). In addition, marginal bone loss was also lower in women and when the opposing dentition was natural teeth, followed, in order, by implant-supported fixed bridges, mixed and implant-supported overdentures.

## Figures and Tables

**Table 1:** Frequencies of the variables included in the study.

Variable						*p* Value
Age (mean (min, max))	58.27 (44, 81)					-
Gender (number of patients)	Women = 18	Men = 17				0.87
Implant location (number of implants)	Mandible = 122	Maxilla = 220				0.09
Maxillary sinus floor elevation (number of implants)	No = 273	Yes = 69				0.001
Implant diameter in mm (number of implants)	3.5 = 94	4 = 96	4.5 = 139	5 = 13		0.001
Implant length in mm (number of implants)	6 = 27	9 = 54	11 = 138	13 = 56	15 = 67	0.001
Abutment height in mm (number of implants)	1 = 40	2 = 177	4 = 93	6 = 32		0.001
Opposing Arch (number of implants)	ND = 51	M = 122	ISFB = 15	ISOD = 154		0.001
Opposing Dentition (number of implants)	ND = 95	RD = 19	ISFB = 203	TSFB = 25		0.001
Follow-up (mean (min, max)) in months	48.10 (0.43, 136.93)					

ND: Natural dentition; M: Mixed; RD: Removable denture; ISFB: Implant-supported fixed bridge; ISOD: Implant-supported overdenture; TSFB: Teeth-supported fixed bridge. For abutment height, opposing arch and opposing dentition, proportions tests were carried out for the lowest category. Note: For abutment height, diameter and length, proportions tests were carried out for the lowest category.

**Table 2:** Descriptive statistics of continuous variables of the study.

Variable	Mean	SE	Median	95% CI
Crown Height (mm)	13.04	0.18	12.54	12.69, 13.39
Crown Implant Ratio	1.464	0.035	1.270	1.396, 1.532
Implants per Bridge (n)	4.819	0.119	4.000	4.585, 5.052
Crowns per Bridge (n)	8.059	0.207	7.000	7.652, 8.465
Bridge Ratio	1.672	0.013	1.670	1.647, 1.679

**Table 3:** Marginal bone level (MBL) in implants placed in patients with previous history of periodontitis for the estimator of predictions at 4108 days after loading.

Variable	Mean	SE	95% CI
MBL Mesial	−0.297	0.040	−0.375, −0.218
MBL Distal	−0.317	0.046	−0.409, −0.226
MBL Average	−0. 307	0.042	−0.389, −0.224

**Table 4:** Abutment height influence on estimated marginal bone level (MBL) (percentage of implants).

Abutment Height (mm)	Ranges of MBL (mm)							N = 342
	<−4	−4 < x < −3	−3 < x < −2	−2 < x < −1	−1 < x < −0.5	−0.5 < x < 0	≥0	
1	7.5	5.0	12.5	12.5	15.0	32.5	15.0	40
2	0	0	2.8	3.4	7.9	57.1	28.8	177
4	0	0	1.1	2.2	2.2	65.6	29.0	93
6	0	0	0	0	0	40.6	59.4	32
% of patients	1	1	3	4	6	54	31	

## Data Availability

The data sets used and/or analyzed during the current study are available from the corresponding author on reasonable request.
